# Modelling the Course of an HIV Infection: Insights from Ecology and Evolution

**DOI:** 10.3390/v4101984

**Published:** 2012-10-04

**Authors:** Samuel Alizon, Carsten Magnus

**Affiliations:** 1 Laboratoire MIVEGEC (UMR CNRS 5290, IRD 224, UM1, UM2), 911 avenue Agropolis, B.P. 64501, 34394 Montpellier Cedex 5, France; 2 Department of Zoology, University of Oxford, South Parks Road, OX1 3PS, Oxford, UK

**Keywords:** HIV, AIDS, modelling, evolution, within-host dynamics, mathematics, computational biology, infection course, virus

## Abstract

The Human Immunodeficiency Virus (HIV) is one of the most threatening viral agents. This virus infects approximately 33 million people, many of whom are unaware of their status because, except for flu-like symptoms right at the beginning of the infection during the acute phase, the disease progresses more or less symptom-free for 5 to 10 years. During this asymptomatic phase, the virus slowly destroys the immune system until the onset of AIDS when opportunistic infections like pneumonia or Kaposi’s sarcoma can overcome immune defenses. Mathematical models have played a decisive role in estimating important parameters (e.g., virion clearance rate or life-span of infected cells). However, most models only account for the acute and asymptomatic latency phase and cannot explain the progression to AIDS. Models that account for the whole course of the infection rely on different hypotheses to explain the progression to AIDS. The aim of this study is to review these models, present their technical approaches and discuss the robustness of their biological hypotheses. Among the few models capturing all three phases of an HIV infection, we can distinguish between those that mainly rely on population dynamics and those that involve virus evolution. Overall, the modeling quest to capture the dynamics of an HIV infection has improved our understanding of the progression to AIDS but, more generally, it has also led to the insight that population dynamics and evolutionary processes can be necessary to explain the course of an infection.

## 1. Introduction

It has been more than three decades that the Human Immunodefficiency Virus (HIV) has reached a pandemic state. The worldwide emergence of this infectious agent coincided with the advent of new modelling techniques in epidemiology, e.g., the basic reproductive number *R*_0_ [1], but also in evolution, e.g., the adaptive dynamics framework [2]. Arguably, this was the first time that so many mathematical approaches have been mobilized to decipher the course of an infection. 

**Figure 1:**
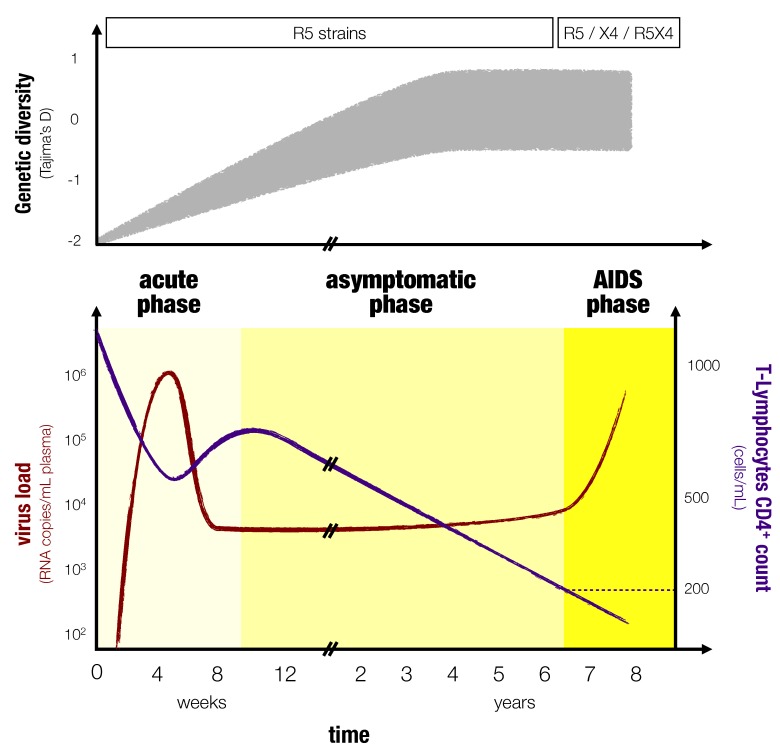
**Typical course of an HIV infection.** The top panel (inspired by [10] and [17]) shows the diversity along with the type of HIV variant that dominates as in [18]. The diversity measure shown here is Tajima’s D, which compares the average pairwise distance of a set of sequences to the number of sites that are polymorphic [17,19]. The bottom panel shows the dynamics of the viral load, in red, and the CD4^+ ^T-cells, in blue as in [3]. The three phases of an HIV infection are stressed with different colors.

Although there can be variations from patient to patient, the course of an HIV infection follows a general pattern [3,4] (Figure 1). The viral load increases exponentially in the first three to six weeks following infection [5,6,7]. The cellular immune response kicks in after one to two weeks followed by the humoral response after four to eight weeks upon infection [8]. This early phase of infection is commonly referred to as the primary infection or initial phase and shares many similarities with acute infections. With the onset of a cellular immune response, the viral load decreases and settles to a more or less constant value for several years. This is the second phase, which is known as the chronic (or asymptomatic) phase. Importantly, even though it might appear as if the virus is resting in this phase, there in fact is a rapid turnover of infected cells and it is the cellular and the humoral immune response that keep viral loads to a constant level, which is referred to as the set point viral load [9]. Furthermore, during this chronic phase, the virus within-host diversity increases [10] and the number of host CD4^+ ^T-cells decreases because they are the primary target of the virus. The third phase or AIDS phase is characterized by a dramatic loss in CD4^+ ^T-cells and a strong increase of viral load (Ho *et al.* [11], Coombs *et al.* [12] showed that viral titers increase in the AIDS phase and O’Brien *et al.* [13], Lyles *et al.* [14] confirmed this trend in longitudinal studies). Clinically, the onset of AIDS is defined as the time point at which the CD4^+ ^T-cell count in the blood falls below 200 per *µ*L. The AIDS phase also often (but not always) coincides with a shift in the virus population and the emergence of virus strains that are able to use CXCR4 co-receptors (instead of CCR5 coreceptors) and thus a wider range of immune cells become susceptible to the virus [15,16]. Because of the fragility of their immune system (low T-cell counts), patients suffer from a variety of opportunistic infections during the AIDS phase. Furthermore, within-host virus genetic diversity tends to decrease during this phase [10,17]. 

An ideal mathematical model of HIV infection should capture the following dynamics. Concerning the virus load, it should exhibit the viral peak in the acute phase, the constant set point viral load during the asymptomatic phase and the viral increase at the end of the infection observed in most HIV infections. Concerning the immune cell dynamics, it should display the decrease of the CD4^+ ^T-cell count in the early acute phase, its slight replenishment to a constant level in the asymptomatic phase and its decrease during the AIDS phase. We refer to these typical patterns as progression to AIDS in the following modeling context. 

The question of how an HIV infection progresses from the acute phase to AIDS has been intensively discussed [20]. Mathematical models had a non negligible part in this debate. It is often thought that the (sole) purpose of mathematical models is to estimate parameters. Indeed, a mathematical model needs to be formulated for estimating parameters associated with the infection, such as the death rate of infected cells or the viral production rate, out of longitudinal data on virus load and T-cell counts. Another use of mathematical models is for comparing hypotheses. If, for instance, there are two competing hypotheses to test, e.g., that virus growth is limited by the availability of target cells or by the immune response [21], one can first estimate parameters for each model and then compare the likelihood of these two models assuming the most likely parameter values for each model [22]. These two uses of mathematical models have been exploited in the case of HIV. However, mathematical modelling is also a tool to investigate potential causative links between observations. This third type of models, often referred to as conceptual models, will be the focus of this review. These models make it possible to extrapolate from well studied and well-understood processes to less well-understood phenomena. For example, a model of virus dynamics can be used to study the consequences of adding a drug that blocks a specific process in the virus life-cycle. As we will see, the case of HIV is quite unique because such conceptual models had a preponderant role in the debate on the cause of AIDS. 

Theoretical models did (and still do) improve our understanding of the course of an HIV infection. For example, Yates *et al.* [23] could show that the so far well accepted hypothesis for the slow depletion of memory CD^+^4 T cells (which they name the “runaway” hypothesis) is only appropriate for the early stages of an infection. The “runaway” hypothesis explains the massive loss of uninfected cells in the chronic phase by homeostatic compensation and/or immune activation of CD4^+ ^T cells, which, as [23] phrase it, would “fuel the fire by generating new susceptible cells and thus more infection” . The problem, as proved by their simple model, is that this hypothesis can only account for a depletion in the range of months and not decades as observed in most HIV infected individuals (the memory CD4^+ ^T cell pool reaches its equilibrium too rapidly). Yates *et al.* [23] conclude that other processes must be at play. More generally, modeling studies have been able to shed a new light on some of the details of the infection but still somehow fail to capture the whole course (and progression) of the infection. Note however that theoretical approaches share this failure with empirical approaches. Interestingly, the inability to explain the course of an HIV infection with a simple model has diverted a lot of the modeling effort towards more specified questions, in particular simple models to estimate parameters (especially related to drug treatments). The reason for this is probably that this is the area where within-host models have proved to be the most useful to clinicians. 

Here, we review mathematical models that have attempted to capture the complete course of an HIV infection. The literature on HIV modelling is plethoric for a glimpse, see e.g., [24,25] but most studies focus on a narrow part of an HIV infection. We mainly restricted our corpus to articles that model HIV infections from the acute to the AIDS phase. Amongst these, we roughly make a distinction between two model categories. A first category of models only involves population dynamics and does not invoke virus evolution to explain the course of the infection. However, simple population models fail to explain the progression to the AIDS phase if they do not include a change of at least one parameter over time. A second category of models studies evolutionary dynamics, *i.e.*, allow for virus evolution and population dynamics to occur. This distinction is not purely arbitrary but, as we will see, they have biological and clinical implications as they reflect the way the infection is understood. More precisely, it is known that HIV evolves over the course of an infection [10], but whether this evolution is only a side effect or on the contrary explains the progression to AIDS remains an open question [26]. We also explore in further details the role of stochasticity in these models, especially with regard to the initial stages of an infection and also with regard to virus evolution. Finally, we mention the role of other within-host processes such as multiple infections and immunopathology and discuss perspectives for future research. 

## 2. Population Dynamics Models

The acute phase (viral peak) and the asymptomatic phase of an HIV infection (set point viral load, stable CD4^+ ^T-cell count) can be captured with a simple target-cell limitation model [25,27]. In particular, this model has led to the estimate of virus replication rates and proves that there is substantial virus replication during the chronic phase [9,28,29]. As shown in Box 1, the model captures the population dynamics of uninfected target cells, infected cells and the virus population with differential equations. The name “target cell limitation” comes from the fact that the production and natural death of target cells lead to an equilibrium level, similar to source-sink models in physics or in ecology. 

The two main discrepancies between this basic model and the typical course of an HIV infection can be seen by comparing Figure 1 and Box 1. First, the target cell density rapidly reaches a set-point value in the basic model. The existence of a set-point in CD4^+ ^T-cell count is not a problem *per se *because if its value is sufficiently low, it can be argued to correspond to the AIDS phase. The key issue is the speed at which the decrease in CD4^+ ^T-cells occurs, which is difficult to reconcile with the slow progression of the disease (see the discussion above and [23]). Second, there is no AIDS phase, *i.e*., once the equilibrium densities of viral load and target cells are reached, they do not vary anymore. Furthermore, Bonhoeffer *et al.* [30] showed that such a model failed to explain the observed dynamics during drug treatment even in the asymptomatic phases: if there was only target-cell limitation, the virus load should be unaffected by the presence of drugs because the decrease in new infections is counterbalanced by the increased availability of susceptible cells. They concluded that another class of models is more likely to explain observations related to drug treatment. In this other class of models, the virus load does not stabilize because of the lack of cells to infect but because infected cells are actively killed by immune cells. Therefore, these models are referred to as “immune control models”. Note that these models in their simplest form also do not account for the increase of the viral load or the decrease in target cell density in the AIDS phase [25,27]. 

As discussed in the following, both the target-cell-limited and immune-limited models of HIV infection have been extended in several ways to account for the complete course of an infection. In this section we focus on models with no (or extremely low) virus diversity such that virus population dynamics are the main driver of the infection and not virus evolution. 

Several models invoke additional cell compartments to account for the course of the infection. For example, Kirschner *et al.* [31] include more realistic immune cell dynamics. T-cells continuously circulate in the lymph and blood and are capable of migrating through the blood vessels into the tissue [32]. Ref [31] incorporate these dynamics into their model by subdividing the T-cell pool into different compartments, both in the blood and the lymphatic system. Each compartment requires an additional differential equation. Only T-cells belonging to specific compartments can produce virions. In addition, there is an exchange of T-cells between some compartments. However, viral load is not integrated specifically into this model and it is only the constant decline in CD4^+ ^T-cells that is interpreted as progression to the AIDS phase.

**Box 1: The target-cell limitation model and a general scheme of HIV within-host models**

The simplest mathematical model to study virus dynamics [27] describes the changes in the density of susceptible target cells (*T*), infected cells (*I*) and free viruses (*V*) with differential equations. Target cells constantly enter the system at rate λ. These cells die at a natural death rate *d_T_* and become infected at rate *β*. Upon infection, cells move into the *I* class and have a potentially increased death rate *d_I_* . Infected cells produce viruses at rate *p*. Viruses are removed from the system at rate *c*. Flow diagrams are a useful tool to illustrate these dynamics. By either solving the system of equations analytically when possible or using numerical methods, we can predict the behavior of densities of viruses and target cells. Stafford *et al.* [33] used this model to estimate the model parameters by fitting the model to viral load data of 10 HIV patients. The figure shows the model dynamics observed when using these estimates (see Table 1).

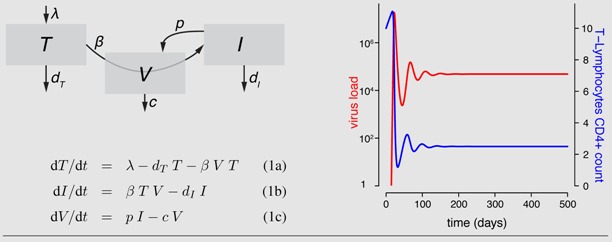

From a mathematical perspective, most of the deterministic, continuous time models are special cases of a general model with *n* different virus populations (*V_j_*, *j* =1 ..., *n*) infecting m different target cell populations (*T_i_*, *i* =1 ..., *m*) leading to infected cells of type *i* with virus *j* (*I_i,j_*). The influx of new target cells is captured by a function *b_i_*(*T_i_*, *I_ij_*), their death by a function *d_T_*_i_ (*T_i_*) and the infection of the cells and thus their transition into the infected class by a function *β*(*T_i_*, *V_j_*, *I_ij_*). Target cells of type *i* infected with viral strain *j *die a natural death according to *d_I_*_i_ (*I_ij_*). However, this death rate can be increased by the immune response (*S_I_* (*I_ij_*, *V_j_*, *T_i_*) or ST (*I_ij_*, *V_j_*, *T_i_*)). Type *j* viruses are produced from infected cells according to *p*(*I_i,j_*) and vanish by death, infection of target cells or different immune functions (*Y_j_*) captured by *c*(*V_j_*, *T_i_*, *Y_j_*). The change in the density of each of the cell and virus types is described by a separate differential equation: 

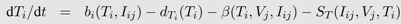
(2a)

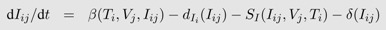
(2b)

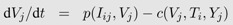
(2c)
The different functions for birth, death, infection and viral production must be adapted to the biological assumptions made in each specific case. The basic target cell-limited model explained above is a special case with *m*, *n* = 1.

**Table 1:** **Mathematical notations used.** This table summarizes all the notations used with their biological description. For parameters, we provide a typical value when it has been estimated (these values are used to obtain the figures). *v* indicates a variable and *f* a function of several variables. Note that for Box 3, Nowak *et al.* [34] do not give units for their rates so we used a dimension week^−^^1 ^(other dimension such as day^−^^1 ^or year^−^^1 ^did not make sense). We also have to point out that some of the parameters used here are still under debate (for instance, recent estimates of the virion clearance rate lead to rates of 5 to 500 day^−^^1 ^[35]).

Symbol	Description	Value
	NOTATIONS USED IN BOX 1	Parameters from [33]
*T *	Density of susceptible target cells	*v*, *T*_0_ = 10 cells · *µ*L^−1^
*I *	Density of cells infected by the virus	*v*, *I*_0_ = 0 cells · *µ*L^−1^
*V *	Density of free viruses	*v*, *V*_0_ = 10^−^^6^ virions · *µ*L^−1^
*b *	Input rate of target cells	*f*, 0.17 cells · *µ*L^−1^ · day^−1^
*β *	Infection rate of target cells by free viruses	6.5 × 10^−^^4^ *µ*L · virion^−1^ · day^−1^
*d_T_*	Death rate of uninfected cells	0.01 day^−1^
*d_I_*	Death rate of infected cells	0.39 day^−1^
*p *	Virus production rate of infected cells	850 virions · cell^−1^ · day^−1^
*c *	Clearance rate of free viruses	3 day^−1^
	NOTATIONS USED IN BOX 2	Parameters from [21]
*Q *	Density of quiescent target cells	*v*, *Q*_0_ = 188 cells · *µ*L^−1^
*r *	Proliferation rate of activated target cells	1 day^−1^
*T_tot_*	= Q + T + I, Total T-cell count	*f*, *T*_0_ = 12 cells · *µ*L^−1^, *I*_0_ = 0 cells · *µ*L^−1^
*T_max_*	Maximal T-cell number	1200 cells · *µ*L^−1^
*V *	Density of free viruses	*v*, *V*_0_ = 10^−^^6^ virions · *µ*L^−1^
*α_Q_*	Activation rate of quiescent T-cells	0.1 − 1 day^−1^
*d_Q_*	Death rate of quiescent T-cells	0.001 day^−1^
*β *	see above	1.35 × 10^−^^3^ *µ*L · virion^−1^ · day^−1^
*γ *	Virus induced depletion rate of activated T-cells	5.6 × 10^−^^3^ *µ*L · virion^−1^ · day^−1^
*d_I_*	See above	0.5 day^−1^
*p *	See above	100 virion · cell^−1^ · day^−1^
*c *	See above	3 day^−1^
	NOTATIONS USED IN BOX 3	Parameters from [34]
*n *	Number of virus strains in the host	*v*, *n*_0_ = 1 strain
*Y_i_*	Density of immune cells specific to virus strain i	*v*, *Yi*(0) = 1 cell · *µ*L^−1^
*Z *	Density of non-specific immune cells	*v*, *Z*(0) = 1 cell · *µ*L^−1^
*r_V_*	Virus replication rate	5 virion^−1^ · week^−1^
*c_1_* and *c_2_*	Activation rates of immune cells	1 cell · virion^−1^ · week^−1^
*u_1_* and *u_2_*	Killing rate of immune cells by viruses	1 virion^−1^ · week^−1^
k_1_	Killing rate of viruses by specific immune cells	5 cell^−1^ · week^−1^
k_2_	Killing rate of viruses by non-specific immune cells	4.5 cell^−^^1^ · week^−1^
*d_1_* and *d_2_*	Baseline death rates of immune cells	0

Perelson *et al.* [36] extend the basic model with target cell limitation by considering uninfected, latently infected and actively infected T-cells. The growth of uninfected target cells has two origins: a constant supply of T-cells from the thymus and a logistic growth term that depends on the total amount of T-cells. Uninfected cells become latently infected T-cells upon infection with the virus and then proceed to the actively infected T-cell class. Only these active cells are able to produce new virus. This model fails to account for the initial peak of virus but it does capture the long term increase in viral load, the decrease in uninfected CD4 T-cell density and the increase in the density of latently and actively infected cells. 

Essunger and Perelson [37] analyse a model with a very detailed description of the T-cell compartment. They distinguish between virgin, activated and memory T-cells. In their model, only activated cells can be infected. Progression to AIDS can then be observed if viral production rate is made time-dependent (which is already a form of evolution). In the same study, a second model allows for infection of resting cells and it can explain the selective depletion of memory cells in the AIDS phase. 

Kirschner [38] and Kirschner and Webb [39] replace the constant birth rate of CD4^+ ^T-cells by a virus-dependent production and proliferation rate of T-cells. Virions are produced by infected T-cells but also by another source of infected cells. This model accounts for the AIDS phase only by increasing the production rate of the non-T-cell based source during disease progression (which again seems to account for evolutionary changes in the virus population). 

De Boer and Perelson [21] study three different model types and they point out that the clinically observed patterns in disease progression cannot be explained in target-cell limited models by infection of CD4^+ ^T-cells alone. However, their activated T-cell model, in which the T-cells are quiescent but can be activated at a certain rate, and where only activated T-cells can be infected, can account for the progression to AIDS if the activation level and/or the viral infection rate increases over time. In their immune-control model, in which CTL effector cells can kill infected cells, progression to AIDS is achieved by changing the activation/proliferation rate over time. Further details about their activated T-cell model can be found in Box 2. 

Fraser *et al.* [40] extend the population dynamics framework by incorporating the slow timescale of resting CD4^+ ^and CD8^+ ^T-cells and the rapid timescale of the turnover of activated CD4^+ ^and CD8^+ ^T-cells. In addition, they add antigenic stimulation, *i.e*., the transition from the resting to the activated class, using a simple random process. This model predicts the general trends in disease progression but also accounts for variability of disease outcomes observed amongst patients. The main drivers of these difference are the efficacy of anti-HIV cytotoxic T lymphocyte responses, the overall viral pathogenicity and additional (non-specified) random effects. In addition, this model is able to predict a variety of responses to anti-viral therapy. 

Finally, one of the most recent examples of models that account for the course of an HIV infection of virus evolution with negligible evolutionary processes is provided by Ribeiro *et al.* [41]. In their model, the progressive decline of one type of target cells leads to a shift in terms of virus target cell preference. This change is very abrupt due to assumptions on functions governed the replenishment of target cells. Purists might argue that there is evolution in a way in their model because they consider three virus types, which differ in the type of cells they can infect and in life-history parameters (infected cell death rate, virus production rate, *etc*.). Since the relative frequency of each virus type varies over the course of an infection, there is evolution *stricto sensu *in the virus population. However, on the other hand, there is no generation of virus diversity at all in their model, which is mainly driven by population dynamics processes.

**Box 2: Activated T-cell model**

In the activated T-cell model by De Boer and Perelson [21], the T-cell pool is subdivided into quiescent and activated T-cells, denoted by *Q* and *T* respectively. This subdivision reflects the finding that HI viruses can infect activated T-cells easier than quiescent T-cells [42]. In the model, quiescent T-cells are activated at rate *α_Q_* and die at rate *d_Q_*. Activated T-cells proliferate at maximal rate *r* but new cells are born into the quiescent T-cell class. Activated T-cells become infected at rate *β*. All the virus-induced depletion of activated CD4^+ ^T-cells is incorporated into the virus induced depletion rate *γ*. Infection of T-cells and virus dynamics are the same as in Equation 1b and 1c. Thus the following system of differential equations describes the activated T-cell model with density dependent proliferation:

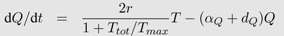
(3a)

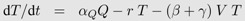
(3b)

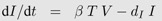
(3c)

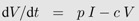
(3c)
The term 2/(1 + *T_tot_*/*T_max_*) acts as a density dependent regulation of the proliferation rate with total T-cell number *T_tot_* = *Q* + *T* + *I* and maximal T-cell number *T_max_*.

The following figure illustrates the model properties. Panel **A** shows that the model with constant parameters cannot predict the progression to AIDS. Theoretically, the maximum T-cell count, the additional depletion *γ*, the infection rate *β* and/or the activation rate of quiescent T-cells *α_Q_* can change over time. However, partial T-cell loss and thus the progression to AIDS is best explained in this framework with increasing activation rate *α_Q_* (**B **and **C**). In **B**, the activation rate is increased by tenfold 400 days after initial infection and in **C**, the activation rate increases linearly over time. All parameter values are taken from [21].

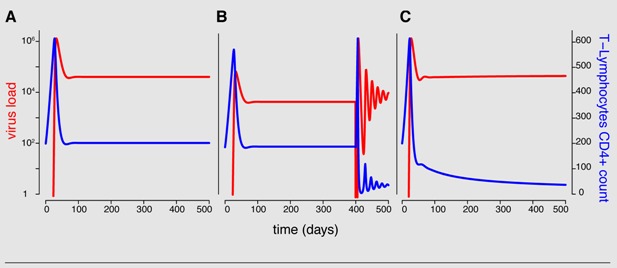

Note that this model is a special case of the general formulation in equation system 2 with two uninfected T-cell types (*m* =2) and one virus type (*n* =1).

## 3. Evolution Models

One of the common features of all the models listed above is that they rely more on population dynamics than on virus evolution to explain the course of an HIV infection. However, this distinction is quite tenuous because accounting for disease progression by changing the viral infection rate or the proliferation/activation rate of a T-cell compartment during the course of an infection [21,38,39] is difficult to justify by another process than virus evolution. We also mentioned that some models, such as the one by [41] considers several virus types that vary in frequency, which can be interpreted as an evolutionary process. Instead of concealing evolution in an implicit formulation or accounting for it in an extremely simplified way, many models have tried to explicitly model virus diversity and evolution. A justification for this is that we do know that HIV infections are diverse and that the virus genetic diversity increases over the course of an infection [10,17,43]. In fact, there is also evidence that virus traits such as the replication rate can evolve over the course of an infection [44,45]. 

One of the first models that included this diversity was one of the most influential, and also one of the most fiercely debated, models in the HIV within-host modelling literature [34]. We extensively describe this model in Box 3 but in a nutshell, its idea is that new HIV strains emerge through mutation and strain specific immune responses fight these new variants. At some point the virus diversity overwhelms the immune response, which leads to a general collapse of the system and to AIDS. By making several simplifying assumptions (e.g., CD4^+ ^T-cell dynamics are not considered), the authors even manage to analytically derive the maximum number of virus strains that an immune system can control, whence the current name of this model: the diversity threshold model. The main factor which drives the progression to AIDS in this model is the asymmetry between the ability of viruses to infect cells and of the immune system to kill viruses: virions can infect all types of target cells but each type of immune system cell can only recognize one particular viral strain. Therefore, each CD4^+ ^target cell has a very small chance to recognize its specific epitope but a high chance of becoming infected. This model by [34] changed the status of ecology and evolution of infectious diseases because for the first time it was argued that evolutionary dynamics could explain the clinical course of an infection. Furthermore, the model leads to patterns that match experimental observations quite well despite relying on only few simple assumptions. Note however that the timing of the onset of AIDS strongly depends on the initial conditions of the model [46]. 

As for the target cell limitation model, several improvements were made to this baseline model. For instance, in a sequel article [47], the authors address the question of what is the fraction of HIV variants that must be recognised by an immunogen (vaccine) to prohibit the development of AIDS. Another model added target-cell limitation to this diversity threshold model and showed that this limited virus diversification [48] (note that in this model, contrary to the [34] original framework, there is no killing of the cells involved in the strain specific immune response by the virus). For completeness, Iwami *et al.* [49] developed a model that also relies on a diversity threshold. Their setting is very similar to that of Regoes *et al.* [48] and they show analytically that there exists an upper threshold in terms of the number of virus strains the immune system can contain.

**Box 3: Diversity threshold model**

In the diversity threshold model by Nowak *et al.* [34], the progression to AIDS comes from the accumulation of HIV strains. One of the key features of this model is that viruses can kill immune cells (at a rate *u_1_* and *u_2_*). The model is based on 2 *n* +1 equations (*n* being the number of HIV strains): one equation for the density of each virus strain (*V_i_*), one equation for the density of each clone of immune cells recognizing this strain (*Y_i_*) and a final equation for the density of non-specific immune cells that can target any virus strain (*Z*) (see Table 1 for further description of the parameters):

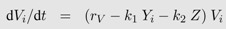
(4a)

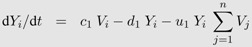
(4b)

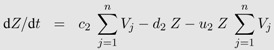
(4c)
The following figure illustrates the predicted viral dynamics, each strain represented by a different color. At each time step, there is a probability *µV_i_* that a cell infected by strain *i* produces a new virus strain. The different panels differ in the mutation rates (**A**: *µ* =1.2 × 10^−^^3 ^, **B **and **C**: µ =1.6 × 10^−^^3^) and the initial conditions (**A **and **B **have the same initial viral load and immune density whereas these are ten times smaller in **C**). The sudden exponential increase in the viral load marks the onset of AIDS (note that the time scales differ in the panels).

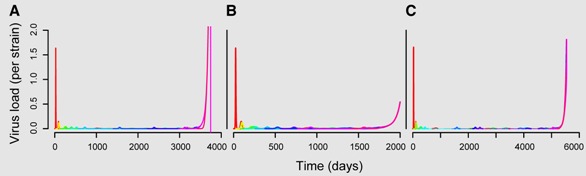

This model nicely captures the virus load dynamics and the increase in virus diversity. However, it has been criticized for mainly three reasons. (i) The mutation process is unclear in the framework and in order to predict the timing of AIDS one needs to assume that mutants emerge at regular time steps. (ii) The initial conditions have an enormous effect on the predictions of the diversity threshold (cf. panels **B **and **C**). (iii) The diversity dynamics do not match those observed *in vivo*. However, this last point of criticism is probably the most fragile because diversity in this model is based on “strains” and not on genetic distances. In order to compare the model to data, it would be necessary to quantify HIV diversity by estimating the number of different epitopes that activate immune responses at a given time.

This model is a special case of the general formulation in equation system 2. Virus production is independent of target cell dynamics and is only captured by the constant viral replication rate *r_V_* . Equations 2a and 2b are omitted and thus it follows *p*(*I_ij_*, *V_j_*)= *r_V_* *V_j_* in Equation 2c. In contrast, immune dynamics are described in much more detail, *i.e.*, the clearance term in Equation 2c equals (*k_1_Y_i_* + *k_2_ Z*) *V_i_*, where *Y_i_* and *Z* are each captured with a separate differential equation.

Whilst the original publications introducing the diversity threshold [34,50] only performed qualitative comparisons between the simulations and the data, other experimental biologists attempted to challenge this hypothesis with clinical data. In particular, Wolinsky *et al.* [51] used longitudinal data of six individuals infected by HIV-1 to show that the rate of CD4^+ ^T-cell loss was not associated with a particular trend in terms of genetic diversity. They also stressed that amino acid changes of the virus were consistent with epitopes being targeted by cytotoxic T lymphocytes, which they interpreted as evidence of adaptive evolution. 

One of the important aspects of the diversity threshold model is that it relies on phenotypic diversity, not on genetic diversity. As Nowak *et al.* [52] point out in their reply to [51]’s study ‘*it remains unclear to what extent genetic variation [. . . ] represents antigenic variation*’. This is because each virus strain is defined by its ability to elicit an entirely specific immune response. This assumption can be criticized from a biological point of view because it means that there is a specific immune response ready to target any possible strain that might emerge [53]. Nevertheless, this assumption also means that virus genetic data as in [10,17] cannot be used to challenge this model because phenotypic diversity is different. Therefore it still remains unclear whether the diversity threshold model goes beyond a qualitative description of virus evolution and whether it can be tested quantitatively. 

Schenzle [54] took another approach to integrate virus evolution in order to explain the course of the infection. The main difference to the diversity threshold model is that [54] modeled the evolution of the virus population in a deterministic way. Instead of introducing different viral strains, his model follows the dynamics of the total virus population (with one differential equation), while the dynamics of one viral trait averaged over the whole virus population (the average CD4^+ ^T-cell infection rate) is captured by a separate differential equation. The implicit assumption made here is that virus replication rate increases over time. A theoretical model by Iwasa *et al.* [55] has shown that, in the absence of trade-offs, the “pathogenicity” of a virus (defined as the inverse of the equilibrium number of target cells) should increase over the course of an infection, provided that there is an accessible evolutionary trajectory. Studies have argued that HIV replication rate increases over the course of the infection [44] and recent data analysis using a predictive algorithm based on the virus sequence has confirmed this result [45]. 

In itself, it is not problematic that the evolution of a life-history trait is captured by an equation. In fact, this is what a recent framework that combines the population genetics and population dynamics does [56] and it can be applied to within-host evolutionary dynamics [57]. However, in the latter approach, usually referred to as the “Price equation” approach, the equations for trait dynamics stem from the genetic diversity of the virus population and from the population dynamics. This is not the case in [54] where the dynamics of the trait is defined arbitrarily. This approach makes it impossible to link the results to other evolution models. Besides the total virus population and the dynamics of the CD4^+ ^T-cell infection rate, Schenzle [54] includes target cells, infected cells and anti-viral activity in his model. Overall, his model can quantitatively describe T-cell depletion due to direct killing by HIV and persistent infection dynamics due to virus evolution. Different incubation periods to AIDS among individuals derive from sensible assumptions concerning the variation of model parameters. Stilianakis *et al.* [58] further study the effect of changing biologically relevant parameters in this model framework, like the increase in virus reproduction rate or the initial values of the basic reproduction number. These parameter changes can account for the different patterns of CD4^+ ^T-cell decline among different patients. 

Stilianakis and Schenzle [59] extend the models presented in [54,58] by subdividing the CD4^+ ^T-cell population into non-susceptible, susceptible and productively infected cells. The fraction of CD4^+ ^T-cells entering the pool of susceptible cells is assumed to be time-dependent. This model has more biological relevance than the other two and explains the whole infection and AIDS phase very well. 

More recently, models have been developed that account for the evolution of virus traits over the course of the infection by describing the dynamics of virus strains with different trait values instead of capturing virus evolution by an (arbitrary) equation. Ball *et al.* [60] use a target-cell limited model that allows for virus diversification. The originality of their model, which allows them to go further in their analyses than earlier models that included differences in virus traits [50], is that they assume a trade-off between the virus replication rate and the death rate of an infected cell. In other words, cells that are more efficient at producing viruses die earlier see also [61]. The interesting pattern captured by the model by [60] is that strains dominating early in the infection differ from strains that dominate late in the infection. The reason for this shift is that the availability of susceptible cells decreases. Therefore, they argue that the progression of an infection could be driven by virus evolution, which itself would come from the target cell dynamics. Recently, Huang *et al.* [62] extended this framework to also include CD8^+ ^T-cells (*i.e*., an immune response acting against the virus). They show that there exist three critical values for the virus replication rate: the first (and lowest) value allows the virus to establish in the host, the second is the threshold that allows the virus to avoid being eradicated by the immune response and the third is the AIDS threshold (overcoming the immune response). Unfortunately, contrary to the setting by Ball *et al.* [60], there is no explicit evolutionary model for the virus replication rate and it is only assumed that the replication rate increases over time, which bears the same limitation as the models described above. 

Alizon and Boldin [63] built a model with a similar trade-off but allowed for cell heterogeneity. They show that the virus population evolves towards an evolutionary branching point, where the monomorphic virus population gives rise to a polymorphic population with some viruses that are adapted to one cell type and other viruses that are adapted to the other cell type. The evolution of virus traits over the course of an infection, and particularly the switch between virus types (or the evolutionary branching), is interesting in the case of HIV because there is a switch in terms of co-receptor usage in the virus population in approximately half of the infections. In fact, before the discovery of these co-receptors, the switch in the virus population was referred to as the “phenotypic switch” because early viruses and late viruses exhibited different properties in cell cultures: late viruses would induce syncytia whereas early viruses would not [15]. 

The co-receptor switch has also been studied using an approach from quasi-species theory. The definition of quasi-species has been put forward to describe rapidly mutating viruses [64]. Because of this high mutation rate, there can be significant transitions from one genotype to another. As a consequence, mutations cannot be considered to be rare events and all the genotypes have to be studied simultaneously in order to estimate quantities such as fitness. The unit of selection therefore shifts from the single genotype to the level of the quasi-species. (One problem with this concept is that there are so many definitions of what a quasi-species is that one could argue that the quasi-species concept has itself become a quasi-species.) Kamp [65] used a combination of different frameworks because the structure of her model is similar to the diversity threshold model but it includes R5 and X4 virus types and the viral growth rate of X4 viruses increases as a function of the density of cross-reactive immune cells, which makes it similar to the [54] model. The main result of [65]’s model is that the co-receptor switch occurs as a consequence of the environment. Sguanci *et al.* [66] used a similar methodology with a target-cell limited model in which several rates (transmission rates and death rates) depend on a variable (the concentration of Tumor Necrosis Factor, TNF) the density of which increases continuously over time. As in earlier models [54,58,59,65], the fact that parameters depend on a variable that increases over time leads to an increase in viral load that mimics that observed in an HIV infection. 

Overall, models with virus evolution seem to have more facility to mimic progression to AIDS. We know that viral genetic diversity increases over the course of an infection and we also know that virus traits can evolve, however, the causality link is still unclear [10,17,43]. Put differently, is the change in the virus population what drives the progression of the infection or is this evolution only a consequence of the progression of the infection (which itself would happen for different reasons)? This is still an open question that virus evolution models have not yet managed to solve [26]. 

## 4. The Role of Stochasticity

Stochastic events play a key role right from the early stages of an infection. Whether the small amount of viruses entering the body can establish an infection is a pure chance event. This is highlighted by the fact that the probability of becoming infected upon one coital act with an HIV-positive partner is estimated to be between 0.1 and 1% [67]. Also, by applying phylogenetic methods to genetic samples of early HIV infection, Keele *et al.* [68] could show that one single strain founded an HIV infection in 80% of HIV transmission via the heterosexual route. 

These stochastic events can be captured with different approaches. One strategy is to analyse a stochastic version of deterministic models using Monte Carlo simulations [69,70,71], the Gillespie algorithm [72,73] or stochastic noise terms (also called stochastic differential equations) [74]. Another strategy is to build a stochastic model from scratch [75,76,77]. All these models account for variation of the virus population between hosts and they allow for extinction of virus in the early phase of an infection. However, the only model that can explain the progression to AIDS is the model by Tan and Wu [69] which is a stochastic version of the models by Perelson *et al.* [36], Schenzle [54]. Overall, most of the stochastic models of HIV dynamics have focused on the initial (acute) phase (the study by Tan and Wu [69] is one of the few exceptions). However, stochasticity also drives evolutionary processes during the chronic (or latent) phase through the mutation process. This is probably best illustrated by a model that explains the progression to AIDS only with evolution and without population dynamics [16]. Ref [16] consider two types of HIV strains, the R5 type that has a high fitness and the X4 type that has an even higher fitness. The passage from one of these strains to the other requires to go through a given number of intermediate mutants, each of which has a fitness lower than 1, meaning that in the long run these intermediate mutants are bound to become extinct within the host. The role of stochasticity is that even though each intermediate mutant will eventually disappear, it can give rise to other mutants in the meantime. Depending on the number of intermediate mutants required and on the exact fitness values of these mutants, the time to switch from an R5 to an X4 strain will vary. This could explain the delay in the progression to AIDS (note that earlier studies had put forward this idea without formalizing it [78]). 

Cellular automaton (CA) models inspired from physics account for stochasticity in a completely different way. One of these CA models that had the most influence is that by Zorzenon dos Santos and Coutinho [79] but, as pointed out in a reply by Strain and Levine [80], it only exhibits the acute infection peak for parameter values that are biologically unrealistic. Interestingly, it seems to be the strong structure of the cellular automaton (e.g., that target cells cannot move) that slows the spread of the virus see also the model by [81]. Cellular automatons have the advantage of allowing the incorporation of spatial aspects, in particular population structure. However, the downside is that, first, understanding the effect of some parameters can be challenging and, second, the way in which the spatial structure is captured might strongly differ from the biology [82]. 

More recently, Lin and Shuai [83] extended the classical CA approach of Zorzenon dos Santos and Coutinho [79] by including more biological realism. They modelled cell and virion movement along a two dimensional lattice and defined rules for meeting events. Thereby they include CD4^+ ^T cells, CD8^+ ^T cells expressing different epitopes, B-cell immunity indirectly by an CD4^+ ^helper dependent immune response and different virions. This biologically more realistic approach reflects the acute, asymptomatic and AIDS phase and confirms earlier findings concerning the diversity threshold from Nowak *et al.* [50]. 

Again, it is not clear how these simulations relate to the course of an HIV infection. Furthermore, several conclusions from these CA models, e.g., the fact that the replenishment rate of target cells matters [81], match that observed in epidemiology when using spatial structured models. On this aspect, more insights could be gained from evolutionary ecology models that study how spatial structure affect the spread and the evolution of the disease at the same time [84]. 

## 5. Other Processes

At the risk of turning this section into a catalogue, we present some hypotheses that have been put forward in mathematical models to account for the course of an HIV infection.

One of these hypotheses is that AIDS is the result of “short-sighted” evolution only optimizing virus fitness at the within-host level, not at the between-host level [78,85]. Therefore, over the course of an infection, viruses continuously adapt to the host, which eventually leads to AIDS. However, these viruses are not good at infecting new hosts and selection occurs upon transmission to favor “less evolved” strains. Interestingly, this hypothesis has gained more support with the recent finding that evolutionary rates seem to be lower at the within-host level than at the between-host level [86,87], which is consistent with a model where viruses that are preferentially transmitted would be stored in latent T-cells and then retrieved [88]. 

Another aspect that has received a lot of attention lately is multiple infection of cells, *i.e*., the fact that the same cell can be infected by (genetically) different virions. This has been known for a long time because recombination is observed for HIV requiring such multiple infections [89], but more recent evidence suggest that they are actually very prevalent among infected cells [90,91]. Some multiple infection models have been used to study virus dynamics, see, e.g., [92] but, in general, they are not really linked to the course of the infection. In fact, most of these models have been used to understand the evolution of drug resistance [93,94]. Recently, however, the realization of the importance of direct cell to cell transmission from multiply infected cells [95] has renewed the interest in these multiple infections. 

A model by Bartha *et al.* [96] suggested that the pathogenesis of an HIV infection could be due to immunopathology [97]. Their idea is that local immune activation is advantageous to the virus inducing it, mainly because it increases the number of susceptible target cells available locally. At the within-host level however, systemic activation is selectively neutral because it indifferently increases target cell supply to any viruses. Finally, this immune activation could have a cost at the between-host level by increasing immune pathogenesis, which would lead to host death, thus shortening the duration of the infectious period. This is an example of conflict between levels of selection that typically occur when evolution occurs at multiple scales [98]. 

Almost all models accounting for the progression to the AIDS phase employing evolutionary dynamics focus on the evolution of viruses. However, immune cells also undergo a constant process of evolution [99]. Galvani [100] models this mechanism and identifies the constant stimulation of production of new T-cells from T-cell precursors as a stimulant for the rise of mutated T-cells. This processes accumulates deleterious mutations over time, which can disturb the lymphocyte regeneration. Viruses can still infect these non-functional cells leading to an uncontrolled rise in viral load. 

Another approach to model the constant co-evolution of the immune system with viral strains is taken by Korthals Altes *et al.* [101]. They capture the immune system evolution by considering HIV-specific CD4^+ ^T-cells with different (randomly distributed) avidity to viruses. Note that viruses are not explicitly modelled but linked to the number of infected cells. With this model, the authors can show that avidity of the CD4^+^-regulated immune response is the main determinant for disease progression rather than the breadth of the immune response. In addition they can identify a link between the avidity of the best clones and the time until the onset of AIDS. 

Finally, Hogue *et al.* [102] analyse the role of dendritic cells in disease progression. Dendritic cells take up antigens from their environment, travel to lymph nodes, present antigens to other cells of the immune system and thereby activate CD4^+ ^and CD8^+ ^T cells [32,103]. When infected by HIV virions, dendritic cells can disperse the virus to other parts of the body [104]. Ref [102] extended the ODE framework of viral dynamics to integrate these characteristics. This framework shows that dendritic cells drive the infection in the early stage of an HIV infection when CD4^+ ^T-cell densities are low. In addition, failure of dendritic cell function is a significant driver of progression to the AIDS phase. Note that Iwami *et al.* [105] also modified their earlier framework [49] to incorporate dendritic cells. The originality of their study is that they use parameter estimates obtained from patient data in [33] and show that variability in these estimates account for variability in the time to AIDS. The downside of their approach is that they assume that one of the value of one parameters (the immune impairment rate) increases deterministically over time. 

## 6. Discussion and Perspectives

The failure to understand the course of an HIV infection based on experimental observations only has led to numerous mathematical models. A widespread view is that such modeling studies are at best redundant because they anyway rely on biological data. The case of the course of an HIV infection offers an interesting counterexample to this statement. If we consider the diversity threshold model [34] for instance, it has caused a paradigm shift in the evolutionary ecology community by putting forward the fact that evolutionary dynamics matters. More precisely, this model showed that the dynamical aspects of evolution at a given level (within-host) can explain emerging properties taking place at another level (the course of an infection and the progression to AIDS). In the end, it is interesting that this framework, which was originally developed to understand HIV infections, has had more influence in the ecology field than in the HIV field. This might be due to the reluctance of their designers to challenge it with longitudinal patient data (in most of their models, they do not go beyond a comparison of general trends but they do not attempt to fit parameters and compare model likelihoods). 

**Figure 2:**
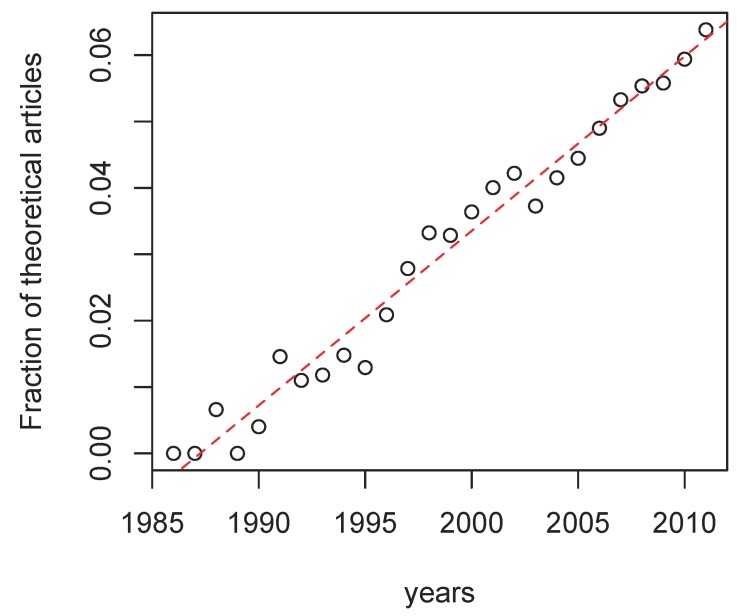
**Fraction of articles on HIV that involve theoretical biology.** The regression was highly significant (*r*=0.0027, p-value < 10^−^^3 ^and adj-*R*^2 ^=0.97). The data was collected on Web of Science on July 13, 2012. The articles on HIV were selected using the keywords Topic=(HIV) AND Topic=(virus OR immunodefic*) and there were 110, 064 hits. The restriction to theoretical articles was performed by adding the keyword AND Topic = (dynamics OR mathemat* OR computational) and there were 4, 277 hits.

Despite a slightly dismissive tendency against mathematical models in the biological sciences in general [106], the fraction of theory-oriented articles on HIV seems to increase linearly over time (Figure 2). However, while writing this review, we were surprised by the strong decrease in the number of articles that fitted our selection criterion (mathematical models of the course of an HIV infection) over time. In fact, the majority of the articles we cite were published in the 1990s. Over the last decade, it has become increasingly difficult to publish models in the field of HIV if they do not analyze (preferentially novel) data. As we pointed out in our introduction, mathematical models of HIV have had a lot of success in parameter estimation. However, this is not always feasible [107,108]. Also, even with simple HIV models, small variations in parameter values can generate large fluctuations in viral load [109]. That models are backed up with biological observations is of course a necessity, however imposing data analysis also tends to restrict the role of mathematical models to parameter estimation at the expenses of conceptual modeling. 

As we have shown here, basic models of virus dynamics can explain the acute and latent phase of an HIV infection but they fail to explain the progression to AIDS (see Table 2 for a summary of existing studies). Only when some parameters changes over time, mathematical models can capture the whole course of an infection. These advanced models allowed researchers to test some hypotheses that can explain the onset of AIDS. We showed that these hypotheses include virus diversity, loss in immune cells, phenotypic switches of the virus, *i.e*., cell tropism or virulence, or multiple cell infections. However, we still do not clearly understand which of these factors or which combination of these factors actually leads to the onset of AIDS *in vivo*. Only an association between experimental and theoretical approaches will eventually allow us to fully understand HIV infections. 

Some important biological facts remained unmentioned in this review. One of these is that multiple infection, *i.e*., the simultaneous infection of the same host by different pathogen species, is increasingly recognized as a major challenge to understand the course of an HIV infection [110,111]. Within-host models that account for these co-infections have been developed for a long time [112,113]. These models argue that other pathogens lead to the activation of helper T-cells, which subsequently can be infected by HIV. If the number of activated T-helper cells exceeds a certain threshold, the target cell pool for HIV virions increases massively. This is followed by infection and destruction of the T-helper cells and the immune system is unable to control pre-established pathogens. This dynamics finally leads to AIDS. 

**Table 2:** **Overview of HIV dynamics models.** We list all the models described in the main text that focus on the course of an HIV infection. For each model, we indicate the number of CD4^+ ^T-cell compartments, the number of virus strains (“*v*” means it varies as the virus evolves and the number of strains is then denoted by *n_v_*), whether the model includes a host anti-viral immune response (and if it does so which type of response) and whether it follows the entire infection and is able to reproduce the slow time scale of CD4^+ ^T-cell decline. We split the CD4^+ ^T-cell compartment into uninfected and infected compartments depending on whether the cells of this compartment are infected with viruses or not. If a paper includes more than one model, we list these models separately (“basic” stands for “basic model”, “act. T” for “activated T-cell model”, ‘im. con.’ for “immune control model” and “drug” for “drug model”). In the models where the number of viral strains are “NA”, the virus dynamics is assumed to be in quasi-steady state with the infected cells, *i.e*., the viral numbers are a function of the number of infected cells. In the models with “NA” numbers of CD4^+ ^T-cell compartments, viruses are assumed to be generated at a constant, target-cell independent rate. If the model captures the progression to the AIDS phase, we list the driving force for disease progression. Here, “NA” indicates that a feature is not included in the model.

Model	Number of compartments:			Immune response	Dynamics captured:		Timescale of asymp-	Driver of disease progression
	uninfected	infected	viral		CD4^+^ T-cells	virus load	tomatic phase	
	CD4^+^ T-cells		strains					
POPULATION DYNAMICS MODELS								
Perelson *et al.* [36]	1	2	1	NA	no initial peak but long-term increase in viruses anddecrease in T-cells		between 3 and 9 years	slow progression due to different T-cell compartments and initial parameter choice
Essunger and Perelson [37]	3	4	1	NA	no initial peak but long-term increase in viruses and decrease in T-cells		between 2 and 8 years	time-dependent viral production rate, initial viral peak observable for model extension allowing infection of resting cells
Perelson *et al.* [29]	1	1	1 and 2 (drug)	NA	acute and asymptomatic phase		∞	only by changing parameters manually during simulations
Kirschner [38]; Kir-schner andWebb [39]	1	1	1	NA	no initial peak but long-term increase in viruses and decrease in T-cells		approx. 4 years	increasing the non-T-cell based viral production rate over time
De Boer and Perelson [21]	1 (basic), 2 (act. T), 0 or 1 (im. con.)	1 (basic), 1 (act. T), 1 (im. con.)	1	CD8^+^ (im cont only)	yes	yes	depending on parameter choice	different models described, progression to AIDS only achievable by changing the activation or proliferation rate in a special T-cell compartment (immune control model) or the viral infection rate (activated T-cell model) over time
Kirschner *et al.* [31]	2	4	NA	NA	no	yes	< 10 years	slow but constant drop of CD4^+^ T-cells due to multi-compartment model
Fraser *et al.* [40]	2	2	NA	CD8^+^	yes	no	4-14 years	slow and fast compartments and (random) antigenic stimulation
Perelson [27]	1	2	1 (basic)	NA	acute and asymptomatic phase		∞	only by changing parameters manually during simulations
Ribeiro *et al.* [41]	2	6	3	NA	yes	yes	approx. 4 years	two virus types using different coreceptors, rise in X4 type due to selection and dominance of X4 virus
EVOLUTIONARY MODELS								
Nowak *et al.* [34]	NA	NA	*v*	general and strain specific	NA	yes	6 to 8 years	antigenic diversity threshold, asymmetry between viral infection and viral recognition
Nowak and May [47]	NA	NA	*v*	general and strain specific	yes	yes	6 to 8 years	diversity threshold and asymmetry, similar model as [34]
Nowak *et al.* [50]	1	1+* n_v_*	*v*	general and strain specific	yes	yes	approx. 8 years	diversity threshold and asymmetry
Schenzle [54]	1	1	1	general	yes	yes	approx. 10 years	within-host evolution modelled by increasing CD4^+^ T-cell infection rate during the infection
Stilianakis *et al.* [46]	NA	NA	*v*	general and strain specific	yes	yes	depending on initial conditions	diversity threshold and asymmetry, similar model as [34]
Stilianakis *et al.* [58]	1	1	1	general	yes	yes	approx. 10 years	increasing CD4^+^ T-cell infection rate during the infection
Regoes *et al.* [48]	1	2*n_v_*	*v*	strain specific	dynamics not shown		NA	adds target cell-limitation to [34]
Stilianakis and Schenzle [59]	2	1	1	general	yes	yes	approx. 10 years	increasing CD4^+^ T-cell infection rate and increasing susceptibility of CD4^+^ T-cells during the infection
Ball *et al.* [60]	1	*n_v_*	*v*	NA	yes	not shown	NA	target-cell limited model and virus diversification with trade-off between the virus replication rate and the death rate of an infected cell
Sguanci *et al.* [66]	*n_v_*	*n_v_*	*v*	TNF	yes	yes	approx. 4 years	target-cell limited, transmission and death rates depend on TNF concentration
Iwami *et al.* [49]	1	*n_v_*	*v*	CD8^+^	NA	NA	NA	AIDS begins when the number of virus strains exceeds a threshold
Iwami *et al.* [105]	1	1	NA	CD8^+^	not shown	yes	variable across patients	increase in immune impairment rate over time (as in [54])
Kamp [65]	NA	NA	*v*	general and strain specific	yes	not shown	NA	diversity threshold, increasing viral growth rate
Alizon and Boldin [63]	2	2	*v*	NA	not shown	yes	approx. 10 years	trade-off between the virus replication rate and the death rate of an infected cell and cell heterogeneity
Huang *et al.* [62]	1	1	1	general	yes	yes	3 to 8 years	deterministic increase in virus replication rate
STOCHASTICITY-DRIVEN MODELS								
Tan and Wu [69]	1	2	1	NA	yes	yes	approx. 10 years	target cell proliferation rate is a decreasing function of viral load (as in [36])
Zorzenon dos Santos and Coutinho [79]	1	2	1	general	yes	yes	approx. 8 years	CA model; infected cells organize themselves into spacial structures
Regoes and Bonhoeffer [16]	NA	NA	*v *	NA	yes	no	5 to 30 years	emergence of mutants strains with different fitnesses
Lin and Shuai [83]	1	1	1	CD8^+^, B-cells indirectly	yes	yes	influenced by viral mutation rate	CA model; spatial structure, virus mutation (asymmetry)
OTHER PROCESSES								
Galvani [100]	4	1	1	CD8^+^, B-cells	yes	yes	approx. 9 years	elevated production of new T-cell clones that accumulate deleterious mutation
Korthals Altes *et al.* [101]	1	1+*n_v_*	NA	strain specific CD4^+^	yes	no	3 - 40 years	avidity of CD4^+^ T-cell response (the lower the avidity is the faster is progression to AIDS)
Hogue *et al.* [102]	2	1	1	CD8^+^, dendritic cells	yes	yes	dependent on parameter change	induced by change of one or more parameters related to infection and viral production or to immune effector functions

Models are needed that take into consideration new theories to explain disease progression. For instance, most of the models presented in this review make a simplifying assumption concerning the location of the infection by assuming that the infection happens in one compartment. This would correspond to a scenario in which the disease dynamics would take place in the blood. However, other body compartments play also important roles in disease progression. Zeng *et al.* [114] show that damage in the lymphoid tissue as a consequence of an infection leads to limited reconstruction of T cells after antiretroviral therapy. Some viral infection models do account for within-host structure but either this structure is extremely simplified (e.g., [63,115]) or the focus of the article is not really on disease progression [116,117,118]. In addition to the spatial structure, recent advances have also been made in understanding how the functioning of the immune response matters to disease progression, many of which are reviewed by Douek *et al.* [119]. For example, the ability of HIV-specific T-cells to be “polyfunctional” (*i.e*., to have the phenotype of both memory and effector T-cells) seems to help control an HIV infection. Some disease progression models do include memory *vs*. effector T-cells [41] but not polyfunctional T-cells. Another striking feature of HIV-specific CTLs (activated CD8^+ ^T-cells) is that they are more prone to programmed death (they tend to overexpress PD-1). The less the T-cells express PD-1, the better the infection is controlled. Finally, another point Douek *et al.* [119] mention is the importance of immune activation and depletion in the gut. This perturbation of the gut mucosa leads to translocation of microbial products into the main circulation of the body [120]. From a modeling point of view, this latter source of pathogenesis is quite different because it involves another actor (the gut flora) that is unrelated to the host, which also brings us back to co-infection frameworks. Integrating these facts into mathematical models of HIV infection might help to capture the different phases of disease progression. In addition, mathematical models might help to identify key factors in disease progression. 

A second major fact we voluntarily ignored in this review is anti-retro-viral treatment, largely because this would require another (and probably longer) review. HIV mutates extremely rapidly and strains that are resistant to at least one drug can arise in almost all treated individuals. Modeling drug treatment had a huge success in determining strategies to reduce the risk of evolution of drug resistant strains and lead to the combination therapy which is widely used in HIV treatment. These models are becoming increasingly important since treated infections could become the majority worldwide. 

HIV within-host models for drug-treatment mainly focus on a certain aspect of the HIV infection. One of their main goals is to devise treatment strategies that are optimal at the within-host level and at the between host level. One crucial focus of this arm of HIV models concerns the evolution of drug-resistant strains. However, the long-term effects of therapeutic interventions on the onset of the AIDS phase are less studied and currently largely not understood. We therefore see a need for models explaining the whole infection even with the advent of massive access to treatment. 
